# Open questions: How many genes do we have?

**DOI:** 10.1186/s12915-018-0564-x

**Published:** 2018-08-20

**Authors:** Steven L. Salzberg

**Affiliations:** Departments of Biomedical Engineering, Computer Science, and Biostatistics, Center for Computational Biology, McKusick-Nathans Institute of Genetic Medicine, Johns Hopkins University, Welch Medical Library, Rm. 107, 1900 E. Monument St, Baltimore, MD 21205 USA

## Abstract

Seventeen years after the initial publicationx of the human genome, we still haven’t found all of our genes. The answer turns out to be more complex than anyone had imagined when the Human Genome Project began.

## The human gene list

It’s hard to overestimate the importance of the human gene list. Thousands of studies rely upon it, including efforts to discover the genetic causes of cancer, complex disorders such as schizophrenia and dementia, Mendelian disorders, and many more. Upon receiving the DNA sequencing results for a sick patient, the first question usually asked is, “what genes are affected?” The very question itself assumes that we know where the genes are—and yet, despite tremendous progress over the past two decades, our knowledge of the human gene catalog is still far from complete.

The primary goals of the Human Genome Project (HGP), which lasted from 1990 until 2003, were to determine both the DNA sequence and “the location of the estimated 100,000 human genes” [1]. Scientists at the time believed that once we had the sequence in hand, we would fairly quickly be able to determine where all the genes were. Subsequent history has proven otherwise: today there are several competing human gene databases, with many thousands of differences among them. And although the number of protein-coding genes has gradually converged, the number of other gene types has exploded.

## What’s a gene?

In order to answer the question of how many genes we have, we must first agree on what we mean by the word “gene”. The definition has evolved ever since Mendel, but the focus as the HGP got under way was primarily on protein-coding genes; i.e., regions of the genome that are transcribed into RNA and then translated to create proteins. However, many genes are noncoding: the HGP’s original paper, in 2001, acknowledged that “thousands of human genes produce noncoding RNAs as their ultimate product,” although the paper itself reported just 706 noncoding RNA genes [2]. For this discussion, then, let us use the following definition of a gene:

*Gene*: any interval along the chromosomal DNA that is transcribed into a functional RNA molecule *or* that is transcribed into RNA and then translated into a functional protein.

This definition includes both noncoding RNA genes and protein-coding genes, and it also groups all the alternative splice variants at a single locus together, counting them as variants on the same gene. It is meant to exclude pseudogenes, which are non-functional remnants of true genes. Admittedly, though, this definition raises the question of what is meant by functional, and a truly comprehensive definition of the term *gene* would likely take many pages to describe.

Using this definition, though, do we have agreement on the number of protein-coding genes? The short answer is no. The human genome began with the assumption that our genome contains 100,000 protein-coding genes, and estimates published in the 1990s revised this number slightly downward, usually reporting values between 50,000 and 100,000. The two initial human genome papers reported 31,000 [2] and 26,588 protein-coding genes [3], and when the more complete draft of the genome appeared in 2004 [4], the authors estimated that a complete catalog would contain 24,000 protein-coding genes. The Ensembl human gene catalog described in that paper (version 34d) had 22,287 protein-coding genes and 34,214 transcripts.

## An expanding number of RNA genes

The invention of RNA-seq in 2008 [5, 6], which was designed to improve our ability to quantify gene expression, also greatly enhanced our ability to detect transcribed sequences, both coding and noncoding. Many of the subsequently discovered noncoding transcripts contained introns, and were quite long, leading them to be called lincRNAs, for long intervening non-coding RNAs, which was later shortened to lncRNAs, dropping the “intervening”. Databases of lncRNAs (and other RNA genes such as microRNAs) have grown dramatically in the decade since, and current human gene catalogs now contain *more* RNA genes than protein-coding genes (Table 1).

**Table 1:** Gene annotations in Gencode, Ensembl, RefSeq, and CHESS

	Gencode^a^	Ensembl^b^	RefSeq^c^	CHESS^d^
Protein-coding genes	19,901	20,376	20,345	21,306
lncRNA genes	15,779	14,720	17,712	18,484
Antisense RNA	5501		28	2694
Miscellaneous RNA	2213	2222	13,899	4347
Pseudogenes	14,723	1740	15,952	
Total transcripts	203,835	203,903	154,484	323,827

Note that despite the many differences shown for Gencode and Ensembl, Gencode is created by merging the Havana manual annotation and the Ensembl automated annotation, and the releases coincide (https://www.gencodegenes.org/faq.html)

^a^Gencode statistics for version 28 from www.gencodegenes.org/stats/current.html as of July 12.2018

^b^Ensemble statistics for version 92.38, which corresponds to Gencode v28, from ensembl.org/Homo_sapiens/Info/Annotation as of July 12, 2018

^c^RefSeq statistics for release 108 from www.ncbi.nlm.nih.gov/genome/annotation_euk/Homo_sapiens/108/ as of July 12, 2018

^d^CHESS statistics for version 2.0 from ccb.jhu.edu/chess as of July 12, 2018. CHESS does not currently include pseudogenes

## A rapidly expanding number of splice variants

RNA-seq revealed another surprise as well: that alternative splicing, alternative transcription initiation, and alternative transcription termination occurred far more frequently than anyone had known before, possibly affecting as many as 95% of human genes [7, 8]. The implication of these findings is that even if we know where all the genes are, we still have considerable work to discover all the isoforms of those genes, and yet more work to determine whether these isoforms have any function or if they just represent splicing errors, as some have argued [9].

## Where are we now?

The challenge of identifying all human genes still confronts us. One problem with the current state of affairs is that for the past 15 years, just two groups have controlled the dominant gene lists: RefSeq, which is maintained by the National Center for Biotechnology Information (NCBI) at NIH, and Ensembl/Gencode, which is maintained by the European Molecular Biology Laboratory (EMBL). Even after all this time—despite much progress—the two catalogs today have hundreds of disagreements between their lists of protein-coding genes, thousands of inconsistencies between their lncRNAs, and multiple categories of genes (e.g., microRNAs and antisense RNAs) where they diverge even further, sometimes not even agreeing on the type of gene (Table 1). The two catalogs are also still evolving; for example, in the past year alone, hundreds of protein-coding genes have been added to or deleted from the Gencode list. These disagreements highlight the ongoing challenge of creating a comprehensive human gene catalog.

The problem of finding all human genes is too important to leave in the hands of just two groups, especially given the lack of agreement in current databases. In 2017, we created a new human gene database, CHESS, that used a massive RNA-seq collection to assemble anew all of the transcripts from a broad survey of human tissues, which is available as a preprint [10]. The CHESS gene set, which adds > 100,000 new gene isoforms and a smaller number of new genes to existing databases, is intended to provide a more comprehensive collection of human genes. By design, it includes all of the protein-coding genes from both Gencode and RefSeq, so that users of CHESS do not have to decide which database they prefer. Its larger number of genes may include more false positives, but we believe the larger set will nonetheless prove very useful, especially to the many studies of human disease that have not yet found a genetic cause. It hardly needs stating that the CHESS gene set, currently at version 2.0, is not yet final and will certainly improve in the years to come.

The bottom line is that we don’t yet know how many genes we have, although we are making progress. Many genes (especially lncRNAs) appear to be highly tissue-specific, and until we survey all human cell types more thoroughly—which may take many more years—we cannot be sure that we have discovered all human genes and transcripts. For most other animal and plant species, we know even less about their gene catalogs, although our knowledge is rapidly improving. Our inability to find a simple answer to the fundamental question of the HGP does not mean we have failed, however. On the contrary, our knowledge of human genes is vastly richer than it was at the outset of the HGP, and technological advances of the past decade provide me with optimism that we will eventually pin down this number.
